# Supplementary material for: Intercomparison of PurpleAir Sensor Performance over Three Years Indoors and Outdoors at a Home: Bias, Precision, and Limit of Detection Using an Improved Algorithm for Calculating PM2.5
Source: Sensors (Basel). 2022 Apr 2;22(7):2755. doi: 10.3390/s22072755 (PMC9002513; doi:10.3390/s22072755)
Supplement: Supplementary file 1 [file sensors-22-02755-s001.zip › sensors-1606090-supplementary.pdf]

sensors

MDPI

Supplementary Materials

Intercomparison of PurpleAir Sensor Performance over Three Years Indoors and Outdoors at a Home: Bias, Precision, and Limit of Detection Using an Improved Algorithm for Calculating $PM_{2.5}$

Lance Wallace

lwallace73gmail.com

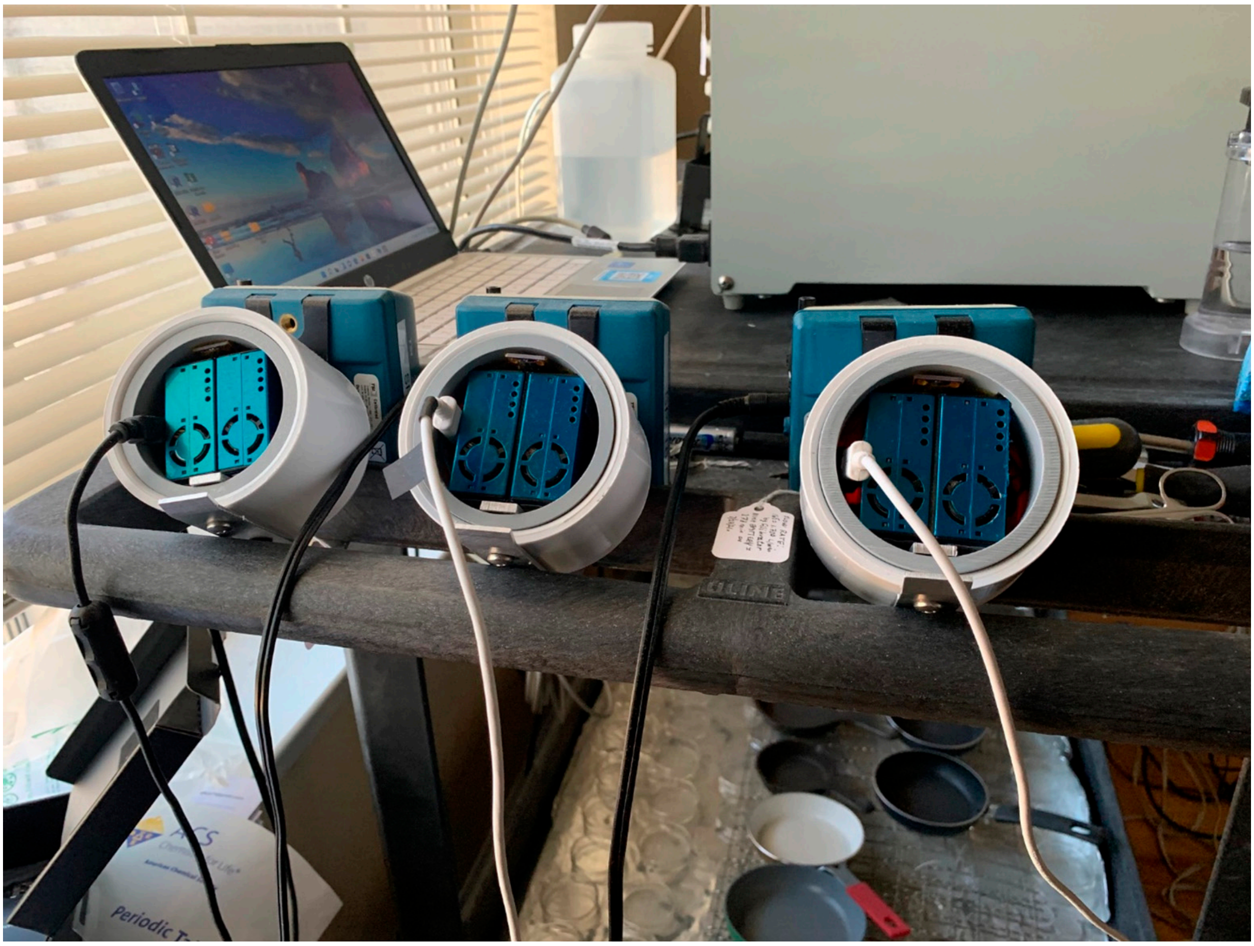

Figure S1. Indoor PurpleAir monitors 1, 3, & 4. These are mounted on a cart and are 1.0 m high. Behind them are three SidePak monitors used as reference instruments during 47 experiments during the 3-year study.

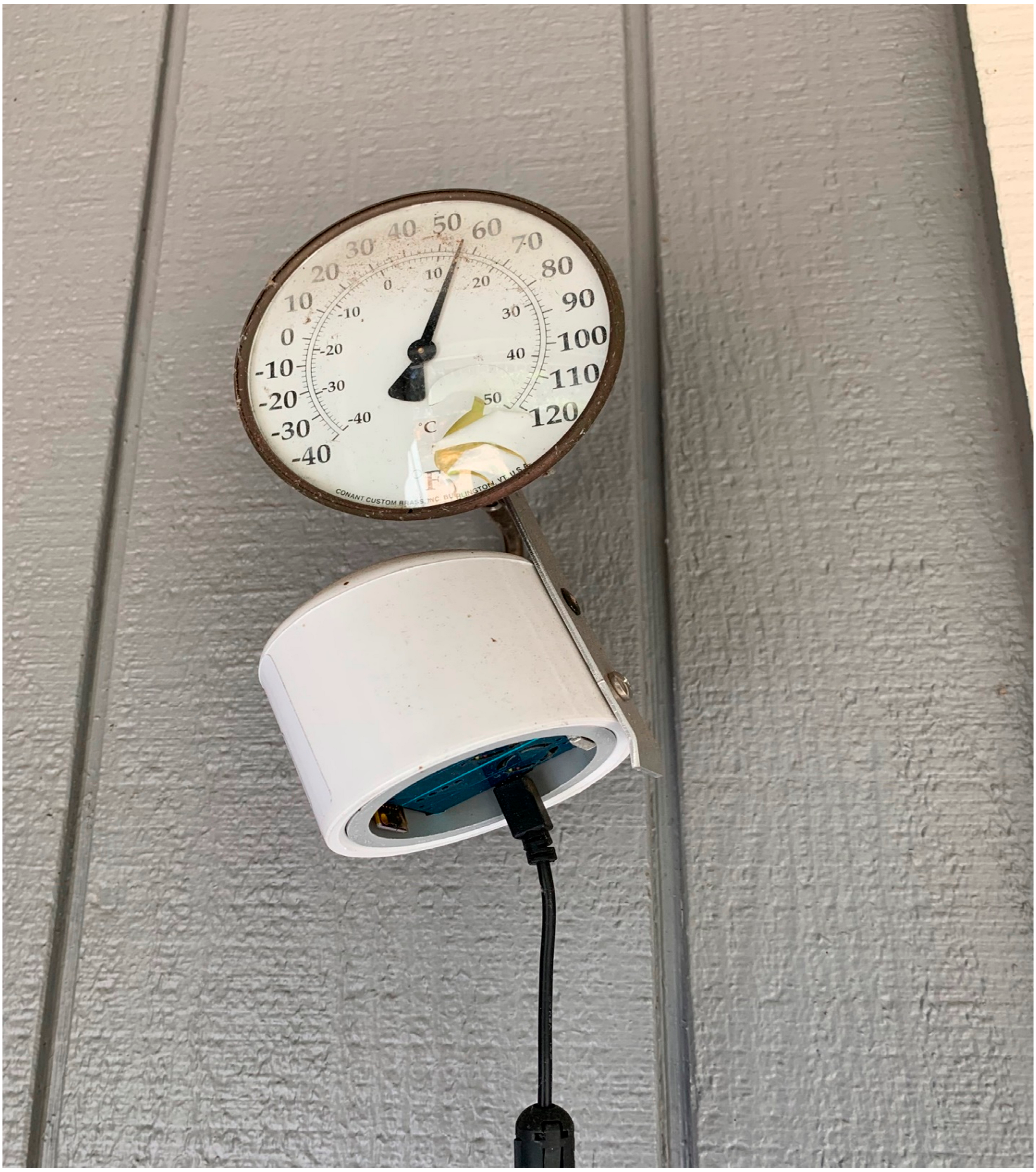

Figure S2. Outdoor PurpleAir monitor attached to bracket mounted 2.0 m from the ground.

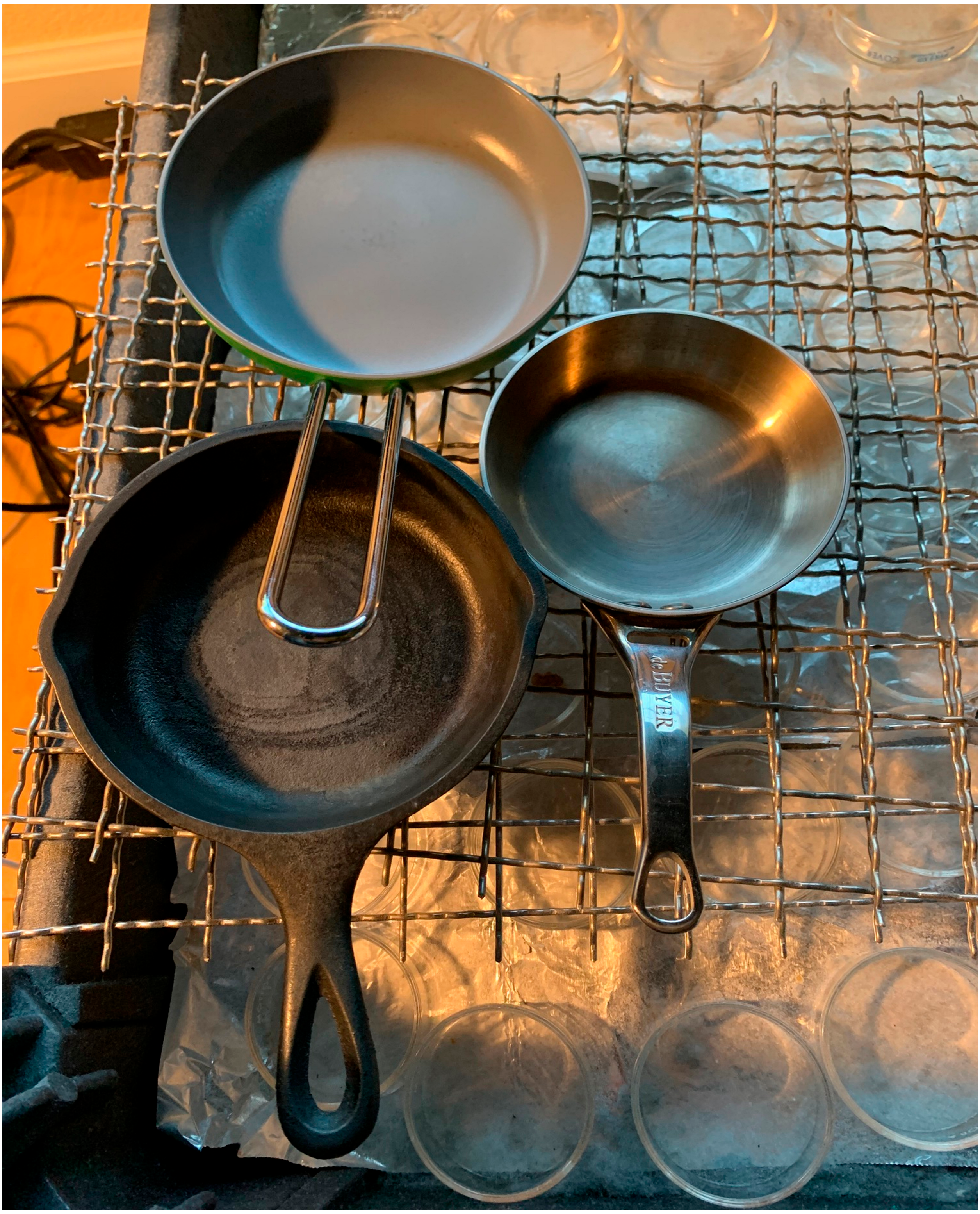

Figure S3. Miniature pans used to heat various foods. Coated pan at top, cast iron at bottom, stainless steel at right.

Figure S4 is the basis for the determination of the calibration factor (CF3) for indoor PA-II PurpleAir monitors. The value of 3.0 for the CF is based on a side-by-side comparison with a research monitor (SidePak) over 47 experiments with an indoor aerosol mixture of PM2.5 created by normal indoor activities together with exhaled breath PM2.5 from vaping liquid marijuana. The SidePak CF of 0.44 was based on 8 gravimetric analyses using a pump-filter combination and a microbalance to weigh the filter. Data shown has been calculated from the original data underlying the peer-reviewed publication: Wallace, L., Ott, W., Zhao, T., Cheng, K-C, and Hildemann, L. (2020). Secondhand exposure from vaping marijuana: Concentrations, emissions, and exposures determined using both research-grade and low-cost monitors, *Atmospheric Environment X*. Volume 8, December 2020, 100093.

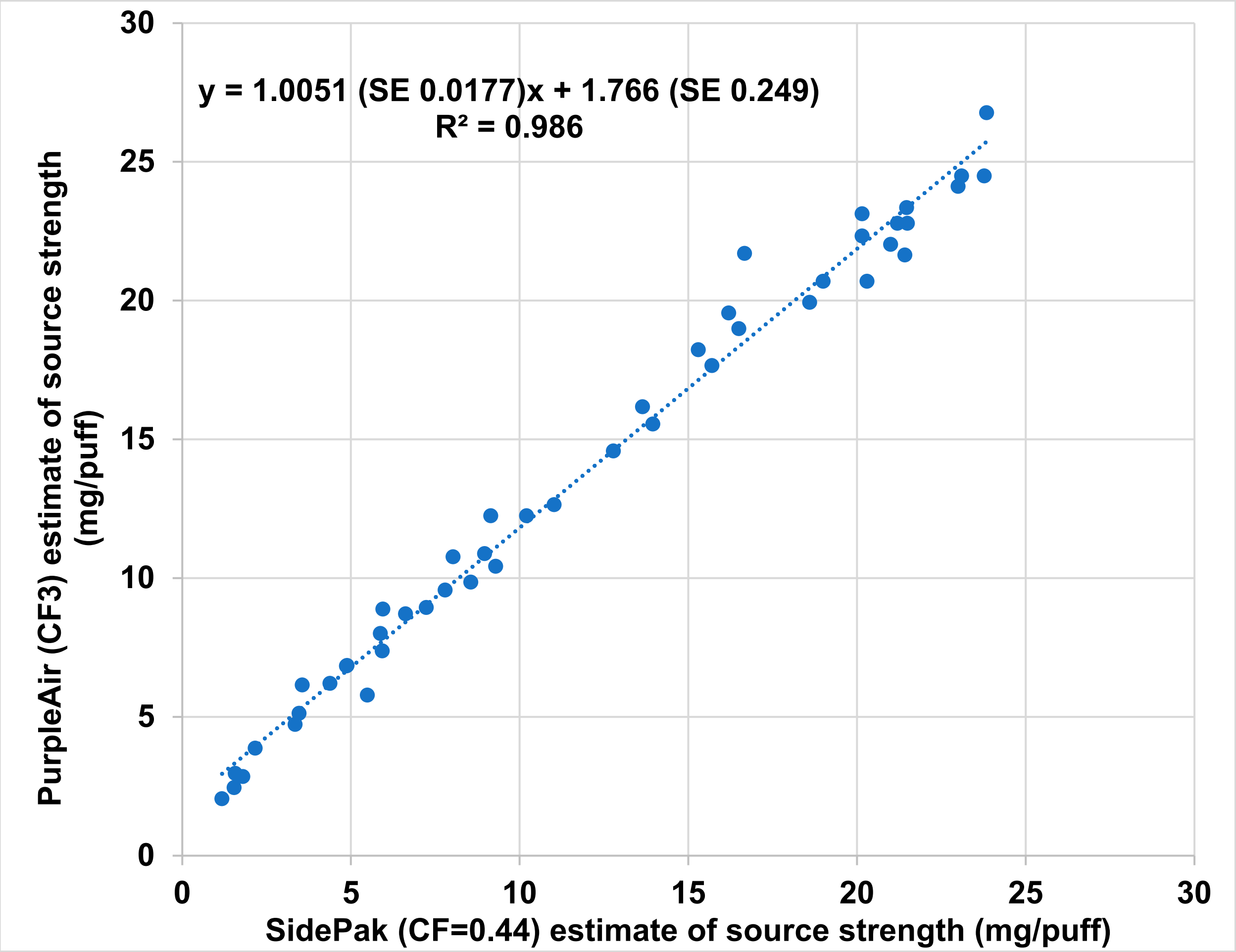

Figure S4. Regression of PurpleAir monitor (CF3) against SidePak monitor (CF0.44). Source: data from Wallace, L., Ott, W., Zhao, T., Cheng, K-C, and Hildemann, L. (2020). Secondhand exposure from vaping marijuana: Concentrations, emissions, and exposures determined using both research-grade and low-cost monitors, *Atmospheric Environment X*, https://doi.org/10.1016/j.aeaoa.2020.100093 (accessd on 2 February 2022).

Table S1. Estimates of PM2.5 concentration (μg/m³) comparing the ALT-CF3 algorithm to the Plantower CF1 algorithm. Time period: 10 January 2019 to 14 January 2022. Monitors 1 & 2.

	Valid N	Mean	Std. Err.	Lower Quartile	Median	Upper Quartile	90th %tile	Max
**PurpleAir ALT-CF3 algorithm**								
Monitor 1 indoors								
1a PM2.5	763102	3.9	0.02	0.64	1.4	2.7	6	1061
1b PM2.5	763102	3.8	0.02	0.63	1.3	2.7	5	1048
Mean 1 PM2.5	763102	3.9	0.02	0.63	1.3	2.7	6	1043
Monitor 2 indoors								
2a PM2.5	499296	4.6	0.03	0.83	1.6	3.0	6	1055
2b PM2.5	499296	4.5	0.02	0.87	1.7	3.0	6	908
Mean 2 PM2.5	499296	4.5	0.02	0.86	1.7	3.0	6	981
Monitor 2 outdoors								
2a PM2.5	242663	4.8	0.02	1.2	2.4	4.4	9	465
2b PM2.5	242663	4.9	0.02	1.1	2.3	4.3	10	473
Mean 2 PM2.5	242663	4.8	0.02	1.2	2.4	4.4	9	469
**Plantower CF1 algorithm**								
Monitor 1 indoors								
1a PM2.5	758867	5.9	0.03	0.2	1.4	3.8	8.6	2312
1b PM2.5	763018	6.0	0.03	0.2	1.4	3.8	8.6	2582
Mean 1 PM2.5	763070	6.0	0.03	0.21	1.4	3.8	8.6	2582
Monitor 2 indoors								
2a PM2.5	493918	7.3	0.05	0.5	1.9	4.3	9.3	2806
2b PM2.5	499251	6.6	0.04	0.1	1.1	3.5	8.6	2300
Mean 2 PM2.5	499275	7.0	0.05	0.31	1.5	3.9	8.9	2521
Monitor 2 outdoors								
2a PM2.5	242541	8.4	0.04	1.3	3.5	7.5	17	791
2b PM2.5	242656	7.4	0.04	0.45	2.5	6.2	16	817
Mean 2 PM2.5	242662	7.9	0.04	0.87	3.0	6.9	17	804

Table S2. Estimates of PM2.5 concentration (µg/m^3) comparing the ALT-CF3 algorithm to the Plantower CF1 algorithm. Time period: 18 June 2020 to 14 January 2022. Monitors 3 & 4.

	N obs.	Mean	SE	Lower Quartile	Median	Upper Quartile	90th %tile	Max
**ALT-CF3 algorithm**								
Monitor 3 outdoors								
3a PM2.5	356484	5.5	0.02	1.2	2.5	4.8	10	421
3b PM2.5	356484	5.9	0.02	1.3	2.6	5.2	10	472
Mean 3 PM2.5	356484	5.7	0.02	1.3	2.6	5.0	10	444
Monitor 3 indoors								
3a PM2.5	42204	7.5	0.08	2.3	3.8	7.9	15	322
3b PM2.5	42204	7.8	0.08	2.3	4.0	8.4	16	316
Mean 3 PM2.5	42204	7.6	0.08	2.3	3.9	8.2	15	319
Monitor 4 indoors								
4a PM2.5	370906	3.5	0.02	0.70	1.5	3.0	6	558
4b PM2.5	370906	3.7	0.02	0.74	1.6	3.3	7	508
Mean 4 PM2.5	370906	3.6	0.02	0.73	1.6	3.1	6	526
**Plantower CF1 algorithm**								
Monitor 3 outdoors								
3a PM2.5	356473	8.4	0.04	1.3	3.3	7.2	16	718
3b PM2.5	356473	9.9	0.04	1.4	3.9	8.5	18	759
Mean 3	356483	9.1	0.04	1.3	3.6	7.8	17	738
Monitor 3 indoors								
3a PM2.5	42184	11.2	0.12	2.7	5.2	12.1	23	506
3b PM2.5	42202	13.1	0.14	3.2	6.1	13.9	27	606
Mean 3	42203	12.1	0.13	3.0	5.6	13.0	25	556
Monitor 4 indoors								
4a PM2.5	370888	5.0	0.03	0.28	1.6	4.1	9	921
4b PM2.5	370897	5.8	0.03	0.35	1.9	4.9	11	891
Mean 4	370904	5.6	0.03	0.33	1.8	4.6	10	891

Table S3. Mean PM2.5 concentrations (ug/m3) and relative bias for the ALT-CF3 algorithm.

	N obs.	Mean	SE	RSE	Bias
1a PM2.5 CF3 IN	353256	3.38	0.016	0.0049	1.031
1b PM2.5 CF3 IN	353256	3.18	0.015	0.0049	0.969
Mean 1 CF3 IN	353256	3.28	0.023	0.0069	
4a PM2.5 CF3 IN	353256	3.65	0.018	0.0049	0.970
4b PM2.5 CF3 IN	353256	3.88	0.019	0.0048	1.030
Mean 4 CF3 IN	353256	3.77	0.026	0.0068	
2a PM2.5 CF3 IN	117804	3.31	0.018	0.0054	0.953
2b PM2.5 CF3 IN	117804	3.64	0.020	0.0055	1.047
Mean 2 CF3 IN	117804	3.48	0.027	0.0077	
2a PM2.5 CF3 OUT	242663	4.82	0.020	0.0042	0.995
2b PM2.5 CF3 OUT	242663	4.87	0.021	0.0043	1.005
Mean 2 CF3 OUT	242663	4.85	0.029	0.0060	
3a PM2.5 CF3 OUT	356484	5.54	0.022	0.0039	0.965
3b PM2.5 CF3 OUT	356484	5.94	0.023	0.0039	1.035
Mean 3 OUT CF3	356484	5.74	0.032	0.0056	

Table S4. Mean PM2.5 concentrations and relative bias for the Plantower CF1 algorithm.

	N obs	Mean	SE	RSE	Bias
1a PM2.5_CF1 IN	242553	7.07	0.040	0.0056	1.012
1b PM2.5_CF1 IN	242553	6.90	0.040	0.0058	0.988
Mean 1 CF1 IN	242553	6.98	0.057	0.0081	
4a PM2.5_CF1 IN	240097	7.54	0.043	0.0057	0.937
4b PM2.5 CF1 IN	240097	8.55	0.049	0.0057	1.063
Mean 4 CF1 IN	240097	8.05	0.065	0.0080	
2a PM2.5_CF1 IN	64344	8.59	0.056	0.0066	0.994
2b PM2.5_CF1 IN	64344	8.70	0.060	0.0069	1.006
Mean 2 CF1 IN	64344	8.65	0.082	0.0095	
2a PM2.5_CF1 OUT	129081	14.00	0.067	0.0048	1.038
2b PM2.5_CF1 OUT	129081	12.98	0.068	0.0053	0.962
Mean 2 CF1 OUT	129081	13.49	0.096	0.0071	
3a PM2.5_CF1 OUT	278256	10.48	0.045	0.0043	0.918
3b PM2.5_CF1 OUT	278256	12.35	0.054	0.0044	1.082
Mean 3 CF1 OUT	278256	11.41	0.070	0.0061	

Table S5. Precision comparing the ALT-CF3 algorithm to the Plantower CF1 algorithm. Time period: 18 June 2020 to 14 January 2022. Monitors 3 & 4.

	N obs.	Mean	SE	Lower Quartile	Median	Upper Quartile	90th %tile	Max
**ALT-CF3 algorithm using precision cutoff at 0.2**								
Monitor 3 outdoors	356484	0.06	7.E−05	0.02	0.05	0.08	0.12	0.20
Monitor 3 indoors	42204	0.04	2.E−04	0.02	0.04	0.06	0.09	0.20
Monitor 4 indoors	370906	0.07	8.E−05	0.03	0.06	0.10	0.14	0.20
**Plantower CF1 algorithm using the ALT-CF3 precision cutoff at 0.2**								
Monitor 3 outdoors	343593	0.16	3.E−04	0.06	0.10	0.17	0.31	1
Monitor 3 indoors	42041	0.10	4.E−04	0.05	0.08	0.12	0.17	1
Monitor 4 indoors	324460	0.20	5.E−04	0.05	0.10	0.21	1	1
**Plantower CF1 algorithm using the CF1 precision cutoff at 0.2**								
Monitor 3 outdoors	278256	0.09	9.E−05	0.05	0.08	0.12	0.16	0.20
Monitor 3 indoors	39011	0.08	2.E−04	0.05	0.08	0.11	0.14	0.20
Monitor 4 indoors	240097	0.08	1.E−04	0.04	0.08	0.12	0.16	0.20
